# Soybean Yield Simulation and Sustainability Assessment Based on the DSSAT-CROPGRO-Soybean Model

**DOI:** 10.3390/plants13172525

**Published:** 2024-09-08

**Authors:** Lei Zhang, Zhenxi Cao, Yang Gao, Weixiong Huang, Zhuanyun Si, Yuanhang Guo, Hongbo Wang, Xingpeng Wang

**Affiliations:** 1 Modern Agricultural Engineering Key Laboratory at Universities of Education Department of Xinjiang Uygur Autonomous Region, College of Water Hydraulic and Architectural Engineering, Tarim University, Alar 843300, China; zhanglei101598@163.com (L.Z.); cy211119@163.com (Z.C.); gaoyang@caas.cn (Y.G.); vooyagee@163.com (Y.G.); 2 Key Laboratory of Tarim Oasis Agriculture, Ministry of Education, Tarim University, Alar 843300, China; 3 Western Agricultural Research Center, Chinese Academy of Agricultural Sciences, Changji 831100, China; 4 Institute of Farmland Irrigation, Chinese Academy of Agricultural Sciences, Xinxiang 453002, China; sizhuanyun@caas.cn; 5 Hubei Key Laboratory of Yangtze Catchment Environmental Aquatic Science, School of Environmental Studies, China University of Geosciences, Wuhan 430078, China; huangwx@cug.edu.cn; 6 Key Laboratory of Northwest Oasis Water-Saving Agriculture, Ministry of Agriculture and Rural Affairs, Shihezi 832000, China

**Keywords:** soybean, DSSAT model, yield, biomass, irrigation water use efficiency

## Abstract

In order to ensure national grain and oil security, it is imperative to expand the soybean planting area in the Xinjiang region. However, the scarcity of water resources in southern Xinjiang, the relatively backward soybean planting technology, and the lack of a supporting irrigation system have negatively impacted soybean planting and yield. In 2022 and 2023, we conducted an experiment which included three irrigation amounts of 27 mm, 36 mm, and 45 mm and analyzed the changes in dry mass and yield. Additionally, we simulated the potential yield using the corrected DSSAT-CROPGRO-Soybean model and biomass based on the meteorological data from 1994 to 2023. The results demonstrated that the model was capable of accurately predicting soybean emergence (the relative root mean square error (nRMSE) = 0, the absolute relative error (ARE) = 0), flowering (nRMSE = 0, ARE = 2.78%), maturity (nRMSE = 0, ARE = 3.21%). The model demonstrated high levels of accuracy in predicting soybean biomass (R^2^ = 0.98, nRMSE = 20.50%, ARE = 20.63%), 0–80 cm soil water storage (R^2^ = 0.64, nRMSE = 7.78%, ARE = 3.24%), and yield (R^2^ = 0.81, nRMSE = 10.83%, ARE = 8.79%). The biomass of soybean plants increases with the increase in irrigation amount. The highest biomass of 63 mm is 9379.19 kg·hm^−2^. When the irrigation yield is 36–45 mm (*p* < 0.05), the maximum yield can reach 4984.73 kg·hm^−2^; the maximum efficiency of soybean irrigation water was 33–36 mm. In light of the impact of soybean yield and irrigation water use efficiency, the optimal irrigation amount for soybean cultivation in southern Xinjiang is estimated to be between 36 and 42 mm. The simulation results provide a theoretical foundation for soybean cultivation in southern Xinjiang.

## 1. Introduction

Soybeans are a significant edible oil crop in China [1]. As a consequence of insufficient domestic production, China has become the world’s first importer of soybeans [2]. It is of great importance to increase the expansion of soybean oil crops and further promote national grain and oil security. Xinjiang is situated in the arid and semi-arid inland region of China, which is rich in land, light, and heat resources. It is therefore an ideal location for the cultivation of soybeans [3]. However, the scarcity of water represents the principal constraint factor limiting agricultural production in this region [4]. The key challenge in ensuring the sustainable development of agriculture in southern Xinjiang is to improve the efficiency with which water resources are utilized and to implement a rational irrigation system.

The continuous expansion of agricultural production has led to an urgent need to address the issue of water shortage. Different degrees of water shortage have been shown to have a significant impact on agriculture [5,6]. One of the most effective ways to ensure high and stable crop yields and save water is through the development of appropriate irrigation systems. This is particularly important in the case of soybean yield. The yield of soybeans was found to be significantly affected by different irrigation amounts. It has been demonstrated that soybeans have high water requirements during the flowering and grain-filling stages. Short-term water shortage does not significantly affect soybean yield, whereas long-term water shortage has a detrimental impact on yield, particularly during the grain-filling period. This is evidenced by the fact that water deficit has a significant impact on soil moisture content [7,8,9]. In a series of field experiments spanning three years, Rajanna and colleagues observed that high soil moisture content extended the growth period of soybean plants. They also demonstrated that an optimal irrigation regimen could enhance soybean yield [10].

Given the complexity of the farmland ecosystem and the limitations of field experiments, the use of crop models to simulate crop growth processes and yields represents an important approach to addressing the impact of climate change and human activities as well as achieving water savings and yield increases in agriculture [11]. A crop growth model is a quantitative and dynamic representation of crop production, informed by the knowledge and research findings of plant physiology, ecology, agro-meteorology, soil science, and other related disciplines. It has been employed extensively in the prediction of crop production potential and the guidance of the irrigation, fertilization, and tillage of agricultural land [12]. The DSSAT-CROPGRO-Soybean model is capable of simulating soybean growth, development, and yield based on genetic parameters interacting with the environment and crop management. Jiang et al. [13] conducted a simulation of the irrigation amount and sowing date of maize in northwestern arid areas. Their findings indicated that the optimized irrigation system could reduce the current irrigation amount by 50%. Guo et al. [14] applied the DSSAT-CROPGRO-Soybean model to correct and validate the genetic parameters of the vegetative phase, reproductive phase, and yield formation process of soybeans at different maturity stages. Additionally, they predicted the soybean yield from 2021 to 2100 under climate change and various technological improvements. Some scholars have also observed that the model accurately simulates crop growth and soil moisture change, but the accuracy of the results decreases with increasing drought [15].

The arid and semi-arid regions of southern Xinjiang are typified by a scarcity of water, high rates of evaporation, and a limited capacity for soil fertility, all of which constrain the potential for agricultural development [16], In the South Xinjiang region, crop cultivation often adopts the planting mode of drip irrigation under the membrane, which not only reduces soil evaporation, maintains soil moisture, and raises soil temperature [17,18,19], but also realizes precise water and fertilizer management, thus increasing crop yields and slowing down the negative impacts of soil evaporation and temperature on the sustainable production of agriculture. Soybeans are a crop with high water requirements, and their production is significantly impacted by water stress. The implementation of rational and effective irrigation strategies is therefore essential to ensure stable yields and enhance irrigation water use efficiency [20,21]. Currently, soybean planting in Xinjiang is primarily focused on the sowing period [22], planting density [23,24], and rhizobia application [25], with relatively few studies addressing the soybean irrigation strategy. Furthermore, there are distinct differences in the management of water and fertilizers in Xinjiang when compared to other regions.

Therefore, in this study, the applicability of DSSAT-CROPGRO-Soybean was calibrated and verified using two-year field trial data from 2022 and 2023. Furthermore, the potential yield and biomass of soybeans were simulated based on meteorological data from 1994 to 2023, and the study analyzed the sustainability and stability of soybean yields under different irrigation strategies for drip-irrigated soybean cultivation under mulch in southern Xinjiang. The aim was to identify suitable irrigation strategies for improving the sustainability and profitability of soybean production and promoting the large-scale production of drip-irrigated soybeans under mulch in arid and semi-arid regions.

## 2. Results

### 2.1. Calibration and Validation of the DSSAT-CROPGRO-Soybean Model

**Figure 1:**
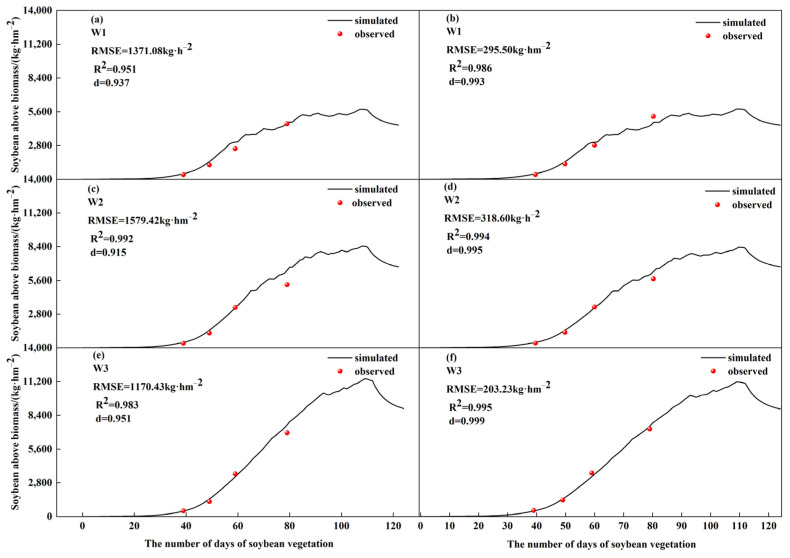
Simulated vs. measured biomass in 2022 and 2023. Note: (**a**,**c**,**e**) refer to the years 2022. (**b**,**d**,**f**) refer to the years 2023.

The simulated and measured values of soybean biomass in 2022 and 2023 are shown in Figure 1 and Table 1. Under different irrigation and fertilization treatments, the soybean biomass normalization index (d) and coefficient of determination (R^2^) were greater than 0.9, indicating that the model has a good simulation effect on soybean biomass. The correction and validation results of soybean phenological period, 0–80 cm soil water storage, yield, and biomass are shown in Table 1. Using the measured data in 2023, we found that the absolute relative error (ARE), relative root mean square error (nRMSE) and R^2^ were 0~22.35%, 0~23.47%, and 0.85~0.99, respectively, indicating that the model simulation was good (ARE < 30%, nRMSE < 30%). The verification of the model in 2022 showed that the simulated values of soybean phenology, 0–80 cm soil water storage, yield, and biomass were in agreement with the measured values (all less than 30%), and ARE, nRMSE, and R^2^ were 0~27.70%, 0~26.34%, and 0.81~0.99, respectively, which further showed that the model had a good simulation effect on soybean growth and yield.

### 2.2. Soybean Biomass, Yield, and Irrigation Water Use Efficiency

**Figure 2:**
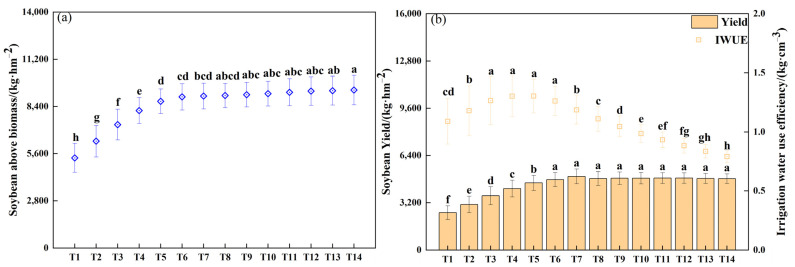
Simulated values of average soybean yield, average biomass, and irrigation water use efficiency from 1994 to 2023. (**a**) soybean above biomass. (**b**) soybean yield and irrigation water use efficiency. Note: T1~T14 represent the irrigation amounts of 24 to 63 mm, respectively. The lowercase letters indicate the difference in significance among treatments at the 0.05 level. The short line represents the standard deviation.

The modified DSSAT-CROPGRO-Soybean model was employed to simulate the average soybean biomass, yield, and irrigation water use efficiency under 14 irrigation amount scenarios from 1994 to 2023 (Figure 2). The results indicated that the soybean yield exhibited a fluctuating pattern with the increase in irrigation amount. The average soybean yield was observed to range from 2529.10 kg·hm^−2^ to 4984.73 kg·hm^−2^. The soybean yield was found to be significantly higher when the irrigation amount was greater than or equal to 36 mm (*p* < 0.05). The highest yield observed was 4984.73 kg·hm^−2^. The biomass of the soybeans increased with the increase in irrigation amount. The average soybean biomass was 5346.97 kg·hm^−2^~9379.19 kg·hm^−2^. The increasing trend of soybean biomass when the irrigation amount was higher than 36 mm was significantly higher than the low irrigation amount treatment (<36 mm). The biomass of soybeans grown with an irrigation amount of 63 mm was the highest at 9379.19 kg·hm^−2^. In a variety of simulation scenarios, the efficiency of soybean irrigation water use is found to be closely correlated with yield change. The mean irrigation water use efficiency for soybeans is 0.79 to 1.30, with the highest efficiency observed at an irrigation amount of 30 to 39 mm (Figure 2b).

### 2.3. Evaluation of Simulated Soybean Yield Sustainability and Stability under Different Scenarios

**Figure 3:**
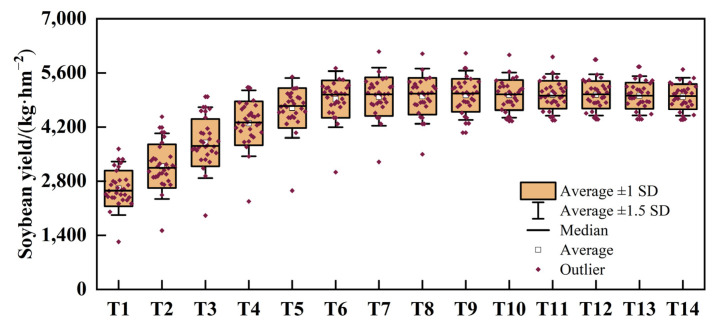
Soybean yield simulation, yield coefficients of variation, and sustainability indices from 1994 to 2023. Note: T1~T14 represent the irrigation amounts of 24 to 63 mm, respectively.

The inter-annual variation, stability, and sustainability index of soybean yields from 1994 to 2023 is presented in Table 2 and Figure 3. The inter-annual variation amplitude of soybean yields exhibits a decreasing trend with an increase in irrigation amount, while the stability index displays an increasing trend with the same increase in irrigation amount. The change trend of the sustainability index is consistent with that of stability. The sustainability index of the T5–T14 yields was greater than 0.7, indicating a high degree of sustainability in soybean production. The variation coefficient of T4–T14 was less than 15%, indicating a high degree of stability in soybean yield change. Comprehensive analysis indicates that the soybean yields under the T5–T7 treatments exhibit high stability and good sustainability.

## 3. Discussion

The DSSAT crop model has been employed extensively to assess the impact of climate change and management strategies on crop growth and yield [26]. In this study, the model was calibrated and verified using soybean phenology, biomass, and yield data from 2022 to 2023. The ARE, nRMSE, and R² were introduced to evaluate the model simulation results. The results demonstrated that the corrected and validated DSSAT model provided a highly accurate simulation of soybean biomass, phenology, and yield. The nRMSE of the phenology, 0–80 cm soil water storage, and biomass was less than 30%, while the nRMSE of the soybean yield was less than 30%. Additionally, the R^2^ values were 0. 81 and 0.85. The mean ARE of the different treatments was less than 30%, which is consistent with the research conclusions of Mulazzani [27], Wang Xingpeng [28], and Du Jiangtao in [29]. This indicates that the model constructed in this paper is a viable approach for simulating soybean growth and yield in southern Xinjiang.

Southern Xinjiang is a region that is typified by irrigated agriculture, and the implementation of moderate irrigation is a fundamental requirement for the achievement of high crop yields. The findings of this study indicate that the response of soybean yield and biomass to irrigation amount differs. The yield exhibits a downward parabolic change trend with an increase in irrigation amount, whereas biomass demonstrates linear growth. This suggests that excessive or inadequate irrigation is detrimental to soybean dry matter accumulation and nutrient absorption and utilization [30]. The yield decreased as a result of the contribution rate of the soybean yield after an irrigation amount exceeding a certain amount. This resulted in more water directly promoting the development of soybeans’ nutritional organs, inhibiting reproductive growth, and ultimately leading to a decrease in yield [31]. Studies have demonstrated that a high irrigation amount of soybean shoot biomass and yield inhibition [32,33], which differs from this study, is largely influenced by regional environmental conditions. The southern Xinjiang area, for instance, is prone to high evaporation and transpiration rates, which have not yet reached the upper limit required to inhibit soybean growth. Consequently, subsequent tests will moderately increase the irrigation amount to verify the aforementioned conclusion. The irrigation water use efficiency is a crucial indicator for assessing the effective utilization of agricultural water resources. The utilization efficiency of irrigation water is negatively impacted by both high and low irrigation amounts, as evidenced by research [34]. The research of Li [35] found that although increasing the irrigation amount can effectively alleviate salinity stress caused by production, it can also cause a significant loss of water and nutrients, which in turn can lead to a reduction in irrigation water use efficiency. Zhang [36] set up 625 different simulation scenarios to examine the impact of increasing and reducing irrigation water on winter wheat. This paper reaches the same conclusion as Zhang [36] regarding the impact of irrigation water on winter wheat.

The sustainability index (SYI) and coefficient of variation (CV) are crucial indicators for measuring the sustainable development and stable yield of crops [37]. This study revealed that the irrigation volume had a significant impact on the sustainability and stability of soybean yields. The low irrigation amount was found to have a detrimental effect on the sustainability and low stability of soybeans. The sustainability and stability of soybean production increases with an increase in irrigation amount. Wang [38] analyzed the results of cotton yields, but too high an irrigation amount inhibits cotton production and increases the waste of water resources. However, increased irrigation under drought conditions promotes increased yields [39]. To ensure the sustainable development of soybeans, it is necessary to choose an appropriate irrigation amount. In addition to the irrigation amount of water, the amount of fertilizer is also an important factor affecting the growth and yield of soybeans. Consequently, the subsequent study will investigate the coupling relationship between irrigation amount and fertilizer amount, with the objective of further promoting the growth and yield of soybeans [40,41]. Meanwhile, only two years of pilot study were considered. In agriculture, trials of more than 3 years may be more convincing, and we will continue to conduct relevant experimental studies. This will provide a theoretical basis and technical support for the appropriate planting of soybeans in southern Xinjiang.

## 4. Materials and Methods

### 4.1. Overview of the Experimental Site

**Figure 4:**
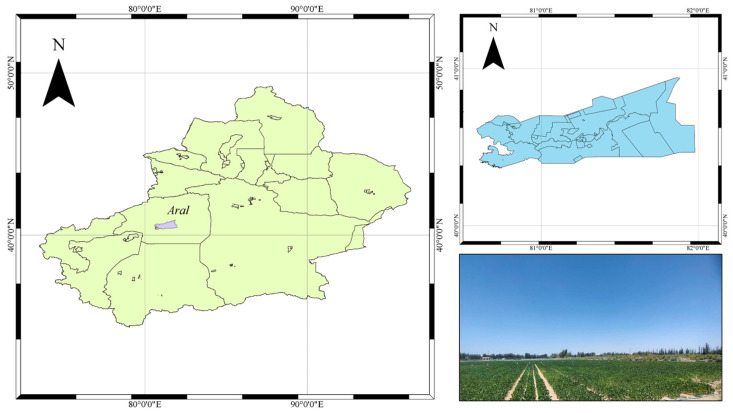
Study area.

The field experiment was conducted in 2022 and 2023 at the Modern Agriculture Field Experimental Base in Xinjiang (81.17′56.52″ E, 40.32′36.90″ N, elevation 1, 100 m) (Figure 4). The area exhibits a typical temperate continental climate, with an average annual temperature of 12.04 °C, annual precipitation of about 46 mm, annual evaporation of approximately 2100 mm, and a groundwater depth of approximately 4 m.

### 4.2. Field Management and Experimental Design

**Figure 5:**
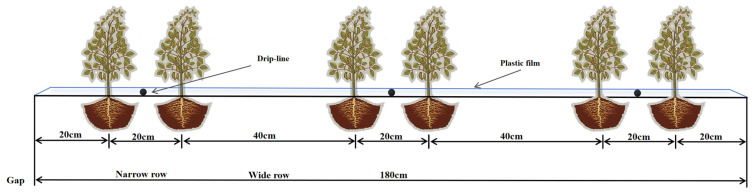
Soybean planting pattern map.

The soybean variety utilized in the field trial was designated as “Tianyou 2986”. Prior to planting, the residential area was levelled. The planting method employed was one film, six lines, and three bands (Figure 5). The wide behavior was 40 cm, the narrow behavior was 20 cm, the plant spacing was 10 cm, and the drip head spacing was 25 cm. The seeding density in 2022 and 2023 was 2.6 × 10^5^ plants per hectare on 20 April and 17 April, respectively. The flowering dates were 29 May and 28 May, and the mature soybean harvest occurred in mid-August. The soil texture in the experimental area is sandy loam, and the average bulk capacity of 0–100 cm is 1.58 g·cm^−3^, and the water holding in the field is 0.24 g·g^−1^; the nitrogen, phosphorus, and potassium levels are 225 kg m^−2^, 180 kg·m^−2^, and 180 kg·m^−2^, respectively.

It was found that the optimal irrigation volume for soybeans in northern Xinjiang was 337–420 mm [42,43], and the optimal irrigation volume for cotton in southern Xinjiang was 406–462 mm [44], whereas less research has been conducted on the irrigation volume of soybean in the southern Xinjiang. For this reason, in the field experiment, three irrigation amounts were applied: 27 mm (W1), 36 mm (W2), and 45 mm (W3). Ten irrigation dates gave a total of 270, 360, and 450 mm of water (Table 3). The amount of nitrogen applied was 225 kg·hm^−2^. Each treatment was replicated three times, with 18 cells in total. The small interval was reserved for shelter rows, and the cell size was 2.33 m by 15 m. Since the soybean with film drip irrigation in southern Xinjiang is still in the preliminary study stage and the *Kc* value has not been determined, this study used meteorological data to calculate the daily water loss, and irrigation was conducted when the accumulated water loss reached 36 mm (*ET*_0_ − P = 36 mm) [35,36]. In the soybean seedling stage, one irrigation was applied to the soybean seedlings, two irrigations were applied during the flowering stage, three irrigations were applied during the pod stage, three irrigations were applied during the grain stage, and one irrigation was applied during the maturity stage, making ten irrigations in total throughout the growth period. Fertilizer was applied with water intervals. The irrigation and fertilization systems for soybeans in 2022 and 2023 are shown in Table 3.

### 4.3. DSSAT-CROPGRO-Soybean Model

The DSSAT model is used to simulate crop growth and development in various environments [45]. The model effectively takes into account all of the parameters in the simulation that directly or indirectly affect soybean seed yield, which mainly includes 18 parameters, and is calibrated and validated using two years of experimental data. The measured data were entered into the DSSAT soybean file, run with the weather and soil files, and the process was repeated until the observed and simulated phenological periods and yields were optimized [46].

#### 4.3.1. Overview of the Model

The DSSAT model includes many plant growth modules, especially on soybeans [47,48], maize [49], and wheat [50]. The DSSAT (v4.8.2) soybean model (DSSAT-CROPGRO-Soybean) was employed to simulate soybean biomass and yield under membrane drip irrigation in southern Xinjiang. The applicability of the model in simulating soybean growth and development and yield in southern Xinjiang was then evaluated. The CROPGRO-Soybean module is employed to simulate the growth and development of soybeans under a range of climatic, soil, and field management conditions. The model input data encompass weather, crop, soil, and field management data [51,52,53]. The soil input data comprise the physical and chemical properties of different deep soil layers. The field management information includes crop varieties, planting date, planting depth, and irrigation and fertilization. Experimental data are entered via the Experimental Data module. It is necessary to create T-files (leaf area index and biomass, etc.) and A-files (phenological period and yield, etc.) in accordance with the standards and formats set out in the DSSAT model.

#### 4.3.2. Meteorological Data

The meteorological data for the period from 2022 to 2023 were primarily obtained from the HOBO meteorological monitoring station installed at the test site. The meteorological data for the period from 1994 to 2021 were obtained from the China Meteorological Science Data Sharing Service Network (http://www.cma.gov.cn/) (accessed on 1 March 2024). The meteorological data required by the CROPGRO-Soybean model encompass a range of variables, including daily maximum temperature (in degrees Celsius), minimum temperature (in degrees Celsius), solar radiation (in megajoules per square meter), rainfall (in millimeters), and others. The data were utilized in conjunction with the WeatherMan program to generate the requisite meteorological documents in the form of WHT files for the model. 

#### 4.3.3. Soil Data

Prior to commencing the field test, it is necessary to sample the initial physical properties of the soil at four depths: 0–20 cm, 20–40 cm, 40–60 cm, and 60–80 cm (Table 4). The SBuild module should be employed to generate the SOL soil files that are necessary for the DSSAT-CROPGRO-Soybean model.

#### 4.3.4. Field Management Data

The field management data primarily comprise seeding date, planting density and depth, and irrigation and fertilization strategies (Table 3). The model field management database was established with the measured field data in 2022 and 2023. The localization application of the DSSAT-CROPGRO-Soybean model necessitates the parameter correction of the varieties. In this paper, the maximum likelihood estimation module (GLUE) in the DSSAT model is employed to validate the phenological period (emergence period, flowering period), biomass, and yield. The output includes the emergence period, flowering period, maturity period, yield, and harvest period, as well as the R^2^ values for the crop varieties (Table 5).

#### 4.3.5. Setting Simulated Scenarios

The quantity of irrigation water applied is a significant factor influencing the growth and yield of soybeans. In order to develop an irrigation schedule that is optimal for the growth requirements of soybeans in southern Xinjiang, a series of 14 simulation scenarios were employed in this study to determine the irrigation amount. The following irrigation amounts were considered: 24 mm, 27 mm, 30 mm, 33 mm, 36 mm, 39 mm, 42 mm, 45 mm, 48 mm, 51 mm, 54 mm, 57 mm, 60 mm, and 63 mm (Table 6). The impact of irrigation amount on potential soybean yield and biomass was evaluated through the use of meteorological data spanning the period from 1994 to 2023. The remaining parameters were aligned with the management protocol employed in the 2023 field experiment.

### 4.4. Aboveground Biomass and Seed Yield of Soybean

From the beginning of the soybean seedling stage, 9 soybean plants were randomly selected in each cell at 15 d intervals, and then disassembled into stems, leaves, and pods. All samples were subjected to drying in an oven at 105 °C for 30 min, after which they were dried in an oven at 65 °C until they reached a constant weight. The dry material weight of each sample was quantified using an electronic balance with a precision of 0.1 g. During the soybean harvest, 2.33 m^2^ quadrats were taken for each treatment, with each treatment being repeated three times in order to determine the seed yield of soybeans and to calculate the yield per unit area.

### 4.5. Irrigation Water Use Efficiency

The calculation of irrigation water use efficiency (IWUE) is as shown in Equation (1).

IWUE = Y/I(1)

where Y is the soybean yield (kg·hm^−2^) and I is the total amount of soybean irrigation water (m^3^·hm^−2^) during the growth period.

### 4.6. Calculation of Soil Water Storage

Soil water storage refers to the volumetric water content at a given soil depth, which can be calculated using the following equation:

(2) $$\mathrm{SW}=\sum_{i=1}^{5}\theta_{i}h_{i}$$

where SW is the soil water storage from 0 to 80 cm (mm), θ_i_ is the soil water content of each layer, and h_i_ is the corresponding soil depth (mm).

### 4.7. Model and Simulation Evaluation Method

The GLUE (generalized likelihood uncertainty estimation) parameter debugging program package was used to determine the parameter rate of crop varieties, and the parameter adjustment number was set to 10,000 times. In this study, the parameters were corrected using data from the 2023 soybean field experiment, and the model was verified and evaluated using data from the 2022 field experiment. In both the model correction and validation processes, the root mean square error between the simulated and measured values (root mean square error, RMSE), nRMSE, ARE, R^2^, and the homing index (normalization index and d) were used for evaluation, and the calculation formula is shown below:

(3) $$\mathrm{RMSE}=\sqrt{\frac{\sum_{i=1}^{n}{{(S}_{i}-M)}^{2}}{n}}$$

(4) $$\mathrm{nRMSE}=\frac{\sqrt{\frac{\sum_{i=1}^{n}{{(S}_{i}-M)}^{2}}{n}}}{\bar{M}}\times 100$$

(5) $$\mathrm{ARE}=\frac{\left|S_{i}{-M}_{i}\right|}{M_{i}}\times 100\%$$

(6) $$R^{2}=\frac{\left[\sum_{i=1}^{n}{(M}_{i}-\bar{M})\times {(S}_{i}-\bar{S})\right]}{\sum_{i=1}^{n}{{(M}_{i}-\bar{M})}^{2}\times \sum_{i=1}^{n}{{(S}_{i}-\bar{S})}^{2}}$$

(7) $$d=1-\frac{\sum_{i=1}^{n}{{(S}_{i}{-M}_{i})}^{2}}{\sum_{i=1}^{n}{\left(\left|S_{i}-\bar{M}\right|+\left|M_{i}-\bar{M}\right|\right)}^{2}}$$

where S is the simulated value, M is the measured value, $\bar{S}$ is the average of the simulated value, and $\bar{M}$ is the average of the measured value. The accuracy of the simulated value is excellent when the nRMSE is <10%, 10% nRMSE is 20%, and 20% nRMSE is 30%. When the nRMSE is >30%, the simulated value is less accurate. The model performs well when nRMSE is 20%, ARE is 30%, and R^2^ and d are close to 1 [26].

### 4.8. Evaluation of Production Sustainability

Simulations were conducted using the DSSAT model. The yield of soybeans was simulated under different irrigation amounts. The sustainability of local soybean production is evaluated by the sustainability index (SYI), while yield stability is expressed by the coefficient of variation (CV). The calculations are as follows:

(8) $$\mathrm{SYI}=\frac{Y_{\mathrm{men}}-\sigma }{Y_{\max}}$$

(9) $$\mathrm{CV}=\frac{\sigma }{Y_{\mathrm{men}}}\times 100$$

where Y_men_ is the mean yield (kg·hm^−2^), σ is the standard deviation of the yield, and Y_max_ is the maximum yield (kg·hm^−2^).

### 4.9. Statistical Analysis

SPSS 22.0 software was used to analyze the difference in the measured data (*p* < 0.05), and Origin 2021 software was used for plotting.

## 5. Conclusions

This study demonstrates the efficacy of the DSSAT-CROPGRO-Soybean model in accurately predicting the phenological period, biomass, and yield of soybeans. These findings suggest that the model can be utilized to simulate the irrigation system of soybeans in southern Xinjiang. The simulation results indicate that when the irrigation amount is 42 mm, the highest soybean average yield is 4984.73 kg·hm^−2^, the average biomass of soybeans is 5346.97 kg·hm^−2^ to 9379.19 kg·hm^−2^, and the average irrigation water use efficiency for soybeans is 0.79 to 1.30. The irrigation water use efficiency at the irrigation amount of 33 and 36 mm is 1.30. When the irrigation amount exceeds 33 mm, it contributes to the sustainability and stability of the soybean yield. In light of the impact of the irrigation amount on soybean biomass, yield, and irrigation water use efficiency, it is recommended that the optimal irrigation amount for soybean planting in southern Xinjiang be set at 36–42 mm, Irrigate ten times throughout the reproductive period: one time in the seedling stage, two times in the flowering stage, three times in the podding stage, three times in the bulging stage, and one time in the ripening stage, with a total irrigation volume of 360–420 mm.

## Figures and Tables

**Table 1:** DSSAT-CROPGRO-Soybean model simulates soybean phenology, biomass, and yield assessment results.

Indicator	Treatment	2022		2023		RMSE		nRMSE/%		ARE/%		R^2^	
		Sim.	Obs.	Sim.	Obs.	2022	2023	2022	2023	2022	2023	2022	2023
Seedling stage	W1	7	7	7	7	0	0	0	0	0	0		
	W2	7	7	7	7					0	0		
	W3	7	7	7	7					0	0		
	Average	7	7	7	7	0	0	0	0	0	0		
Anthesis	W1	35	36	46	44	1	2	2.79	4.55	2.78	4.55		
	W2	35	36	46	44					2.78	4.55		
	W3	35	36	46	44					2.78	4.55		
	Average	35	36	46	44					2.78	4.55		
Maturity	W1	117	114	122	118	3.7	4.61	3.24	4.04	2.63	3.39		
	W2	118	114	122	118					3.51	3.39		
	W3	118	114	124	118					3.51	5.08		
	Average	118	114	123	118	3.7	4.61	3.24	4.04	3.22	3.95		
Biomass	W1	4104	4938	2423	2421	1371.08	295.5	27.76	12.21	28.46	9.56	0.951	0.986
	W2	4155	5591	2901	2701	1579.42	318.60	28.25	11.80	31.49	10.63	0.992	0.994
	W3	4146	5086	3178	3185	1170.43	203.23	23.01	6.38	23.14	3.34	0.983	0.995
	Average	4135	5205	2834	2769	1373.64	272.44	26.34	10.13	27.70	7.84	0.975	0.992
0–80 cm soil water storage	W1	150	155	123	102	15.68	29.66	8.61	23.91	3.22	20.59	0.635	0.721
	W2	186	188	134	133	12.78	15.86	7.02	12.79	1.06	0.75	0.639	0.770
	W3	190	202	148	137	14.02	13.79	7.70	11.12	5.45	8.03	0.650	0.859
	Average	175	182	135	124	14.16	19.77	7.78	15.94	3.24	9.79	0.641	0.783
Yield	W1	3525	3665	1866	2920	450.57	906.2	10.83	23.47	3.82	36.1	0.81	0.85
	W2	3906	4210	3085	4236					7.22	27.17		
	W3	3893	4598	4261	4428					15.33	3.77		
	Average	3775	4158	3071	3861	450.57	906.2	10.83	23.47	8.79	22.35	0.81	0.85

Note: Maturity refers to the number of days until soybeans reach harvest; above biomass refers to leaves, stems, and pods; Yield refers to the yield of the seed at the time of the soybean harvest; Sim refers to simulated value; Obs refers to measured values.

**Table 2:** Soybean yield coefficients of variation and sustainability indices from 1994 to 2023.

Treatment	CV	SYI	Treatment	CV	SYI
T1	0.60	18.25	T8	0.72	9.81
T2	0.56	18.34	T9	0.73	8.75
T3	0.61	16.61	T10	0.74	8.00
T4	0.69	13.67	T11	0.75	7.39
T5	0.73	11.35	T12	0.76	7.30
T6	0.75	10.15	T13	0.78	6.97
T7	0.73	10.02	T14	0.79	6.78

Note: CV and SYI represent the variation coefficient and sustainability index, respectively.

**Table 3:** Irrigation and fertilization regimes for soybean fertility in 2022 and 2023.

Planting Date		Date of Irrigation		Irrigation Amount/mm			Nitrogen Application/kg·hm^−2^	Planting Density/Plants·hm^−2^	Planting Depth/cm
2022	2023	2022	2023	W1	W2	W3			
20-April	17-April	12-May	18-May	27	36	45	0	2.6 × 10^5^	2
		31-May	29-May	27	36	45	45		
		8-June	9-June	27	36	45	0		
		15-June	16-June	27	36	45	45		
		23-June	23-June	27	36	45	0		
		1-July	30-June	27	36	45	45		
		8-July	7-July	27	36	45	0		
		16-July	14-July	27	36	45	45		
		22-July	21-July	27	36	45	0		
		28-July	28-July	27	36	45	45		
		Total amount/mm		270	360	450	225		

**Table 4:** Physical properties of the soil in the test area.

Depth/cm	Clay/%	Silt/%	Sand/%	Wilting Point (g·g^−1^)	Field Capacity (g·g^−1^)	Saturated Water Content (g·g^−1^)	Bulk Density (g·cm^−3^)
0~20	2.43	41.49	56.08	0.10	0.21	0.24	1.60
20~40	2.56	41.40	56.04	0.10	0.24	0.30	1.55
40~60	2.88	42.82	54.30	0.12	0.25	0.33	1.58
60~80	2.60	41.40	56.00	0.13	0.25	0.32	1.59

**Table 5:** Plant Genetic Parameters.

Crop Genetic Parameters	Parameter Description	Value	Value Range
CSDL	Critical short-day length below which reproductive development progresses with no daylength effect (for short-day plants) (hour)	14.01	11.78~14.60
PPSEN	Slope of the relative response of development to photoperiod with time (positive for short-day plants) (1/hour)	0.262	0.129~0.385
EM-FL	Time between plant emergence and flower appearance (R1) (photothermal days)	17.83	9~28.9
FL-SH	Time between first flower and first pod (R3) (photothermal days)	5.5	5~10
FL-SD	Time between first flower and first seed (R5) (photothermal days)	13.34	11~22
SD-PM	Time between first seed (R5) and physiological maturity (R7) (photothermal days)	31.86	22~37
FL-LF	Time between first flower (R1) and end of leaf expansion (photothermal days)	13	11~19
LFMAX	Maximum leaf photosynthesis rate at 30 C, 350 vpm CO_2_, and high light (mg CO_2_·m^2^·s^−1^)	1.054	1~1.4
SLAVR	Specific leaf area of crop under standard growth conditions (cm^2^·g^−1^)	370.6	300~400
SIZLF	Maximum size of full leaf (three leaflets) (cm^2^)	199	137~230
XFRT	Maximum fraction of daily growth that is partitioned to seed + shell	1	1
WTPSD	Maximum weight per seed (g)	0.169	0.15~0.19
SFDUR	Seed filling duration for pod cohort at standard growth conditions (photothermal days)	25.28	17~25.5
SDPDV	Average seed per pod under standard growing conditions (grain/pod)	1.82	1.7~2.44
PODUR	Time required for crop to reach final pod load under optimal conditions (photothermal days)	10.5	10~12
THRSH	Threshing percentage. The maximum ratio of (seed/(seed + shell)) at maturity. Causes seeds to stop growing as their dry weight increases until shells are filled in a cohort.	77	77~78
SDPRO	Fraction protein in seeds (g(protein)/g(seed))	0.405	0.400~0.425
SDLIP	Fraction oil in seeds (g(oil)/g(seed))	0.205	0.200~0.225

**Table 6:** Scenario simulation processing.

Treatment	Irrigation Amount mm	Treatment	Irrigation Amount mm
T1	24	T8	45
T2	27	T9	48
T3	30	T10	51
T4	33	T11	54
T5	36	T12	57
T6	39	T13	60
T7	42	T14	63

## Data Availability

Data will be made available upon request.
